# Hypermethylation of *Secreted Frizzled Related Protein 1* gene promoter in different astrocytoma grades

**DOI:** 10.3325/cmj.2018.59.213

**Published:** 2018-10

**Authors:** Anja Kafka, Valentina Karin-Kujundžić, Ljiljana Šerman, Anja Bukovac, Niko Njirić, Antonia Jakovčević, Nives Pećina-Šlaus

**Affiliations:** 1 Department of Biology, University of Zagreb School of Medicine, Zagreb, Croatia; 2 Laboratory of Neuro-oncology, Croatian Institute for Brain Research, University of Zagreb School of Medicine, Zagreb, Croatia; 3 Department of Pathology and Cytology, University Hospital Center Zagreb, Zagreb, Croatia; * AK and VKK contributed equally

## Abstract

**Aim:**

To identify the involvement of *Secreted Frizzled Related Protein 1* (*SFRP1*) promoter hypermethylation in different malignancy grades of astrocytoma and assess its association with beta-catenin, lymphoid-enhancer factor 1, and T-cell factor 1.

**Methods:**

Twenty-six astrocytoma samples were collected from 2008-2015. Promoter hypermethylation was evaluated by methylation-specific polymerase-chain-reaction and protein expression by immunohistochemistry and stereological analysis. The staining intensity was scored by comparing immunoreactivity with normal tissue and by using 10% and 50% cut-offs.

**Results:**

*SFRP1* promoter methylation was found in 32% of astrocytomas. The number of hypermethylated samples increased in higher astrocytoma grades and was the highest in glioblastoma (*P* = 0.042 compared to other astrocytoma grades). There was 45.8% of samples with the lack of or weak expression of SFRP1 protein and 29.2% with strong expression. Samples with methylated promoter expressed significantly less SFRP1 than samples with unmethylated promoter (*P* = 0.031). Beta-catenin expression levels were elevated. Yet, glioblastomas with unmethylated *SFRP1* promoter had significantly less beta-catenin (*P* = 0.033). Strong expression of lymphoid-enhancer factor 1 was associated to higher astrocytoma grades (*P* = 0.006).

**Conclusion:**

*SFRP1* gene was epigenetically silenced in glioblastomas when compared to low astrocytoma grades, which may suggest that the lack of its protein is involved in astrocytoma progression.

Astrocytomas are the most common primary brain tumors. According to the World Health Organization (WHO), they are classified into four malignancy grades on the basis of their histology and prognosis (1,2). The most aggressive and highly malignant tumor is glioblastoma multiforme (GBM), a grade IV astrocytoma, with survival shorter than a year. For this molecularly very complex and heterogenic tumor, effective therapy is still missing. While pilocytic astrocytomas (grade I) are considered benign, diffuse and anaplastic astrocytomas (grades II and III) can progress to secondary GBM, probably because of incomplete neurosurgical resection. Histological examination cannot precisely predict how these variable tumors will behave clinically or how fast the recurrence will occur (3-5). Since histologically similar tumors may differ in their molecular phenotypes, the classification of brain tumors has recently included genetic profile as an additional criterion (2,6). Therefore, mutation of the *isocitrate dehydrogenase 1* (*IDH1*) gene, named R132H, present in diffuse astrocytoma became an important element for the new WHO classification (7,8).

Wnt signaling pathway functions in embryonic development and maintains adult tissue homeostasis (9,10). However, this pathway is also one of the key oncogenic pathways in human malignancies (11,12). It is associated with many human cancers and is implicated in gliomagenesis (13-22). Wnt signaling is usually malfunctioned by preventing beta-catenin's degradation in the destruction complex, which elevates its cytosolic levels. The molecule is then transferred to the nucleus, where it binds to its transcription partners – lymphoid-enhancer factor/T-cell factor (LEF/TCF). This molecular interaction transcriptionally stimulates a number of promoters of target genes involved in oncogenic transformation (23).

An important regulatory role in Wnt signaling is played by Secreted Frizzled Related Protein (SFRP) family of genes, which codes for proteins that usually limit the pathway’s activity (24-27). The suppression of the Wnt signaling by SFRP gene family contributes to normal development of astrocytes (28,29). Another mechanism involved in astrocytic tumorigenesis is disrupted epigenetic regulation, resulting in the silencing of a great variety of genes (30). A common mechanism for the transcriptional silencing of tumor suppressor genes is promoter hypermethylation (31). *SFRP1* promoter hypermethylation and the loss of SFRP1 protein expression have been reported in tumorigenesis of many human cancers, but their involvement in astrocytoma progression still needs to be elucidated (1,32-34).

We believe that SFRP1 plays an important role in astrocytic brain tumors and that its expression levels, regulated by methylation mechanisms, change according to malignancy grades. Also, we hypothesized that SFRP1 expression would affect Wnt signaling activity. The aim of this study was to identify the status of *SFRP1* promoter hypermethylation in different malignancy grades of astrocytoma in order to better understand the molecular features and offer potential biomarkers. We also assessed the association of its expression levels with beta-catenin, TCF1, LEF1, and demographic data.

## Materials and methods

### Astrocytoma samples

Twenty-six astrocytoma samples were collected from the Departments of Neurosurgery and Pathology of the University Hospital Center (UHC) Zagreb and UHC *Sestre Milosrdnice*, Croatia from 2008-2015. The tumors were identified by magnetic resonance imaging in different cerebral regions. The patients had no family history of brain tumors or familial tumor syndromes, and the diagnosis was made by a board-certified neuropathologist and classified according to WHO guidelines (2). There were 6 diffuse astrocytomas with WHO grade II (AII; 5 male and 1 female patients), 6 anaplastic astrocytomas with WHO grade III (AIII; 4 male and 2 female patients), and 14 glioblastomas with WHO grade IV (GBM; 6 male and 8 female patients). The tumors were newly diagnosed, and patients did not receive any treatment prior to surgical resection. Our group of glioblastoma patients was also tested for the presence of *IDH1*-R132H (monoclonal antibody Dianova IDH1-R132H Clone H09, Dianova, Hamburg, Germany) and ATRX Chromatin Remodeler (*ATRX*) mutations (polyclonal antibodies Sigma HPA001906, Sigma, Taufkirchen, Germany). Fifteen patients were male and 11 were female. Patient age ranged from 24 to 77 years (mean 51.9 years; median 54.5 years). Ethical approval was received from the Ethics Committees of the Medical School University of Zagreb (Case number: 380-59-10106-14-55/147; class: 641-01/14-02/01, July 1, 2014), University Hospital Center *Sestre Milosrdnice* (number: EP-7426/14-9, June 11, 2014), and University Hospital Center Zagreb (number: 02/21/JG, class: 8.1.-14/54-2, June 23, 2014). The patients signed the informed consent for research participation and data presentation. The reference tissue was autologous normal blood DNA.

### DNA extraction

Collected tumor tissues were frozen in liquid nitrogen and kept at -80°C. Peripheral blood samples were collected in EDTA and immediately processed. Approximately 0.5 g of tumor tissue was homogenized with 1 mL extraction buffer (10 mM TrisHCl; 0.1 M EDTA; 0.5% sodium dodecyl sulfate, pH 8.0) and incubated with 100 mg/mL proteinase K (Sigma-Aldrich, St. Louis, MI, USA) overnight at 37°C. Phenol/chloroform extraction and ethanol precipitation followed (35). Blood was used to extract leukocyte DNA; 5 mL of blood was lysed with 15 mL of RCLB (red blood cells lysis buffer; 155 mM NH_4_Cl; 0.1 mM EDTA; 12 mM NaHCO_3_) and centrifuged (15 min/5000 × g) at 4°C. The pellet was further processed in the same way as for DNA extraction from the tissue samples.

### Methylation-specific polymerase chain reaction analysis

DNA isolated from glioblastomas (14 samples), grade II astrocytomas (6 samples), and grade III astrocytomas (6 samples) was treated with bisulfite using MethylEdge Bisulfite Conversion System (Promega, Madison, WI, USA) according to manufacturer’s instructions. Bisulfite-treated DNA was used for methylation-specific polymerase chain reaction (MSP). Primer sequences for MSP of the *SFRP1* promoter region were synthesized according to Guo et al (36): methylated primers, F:5′TGTAGTTTTCGGAGTTAGTGTCGCGC3′ and R:5′CCTACGATCGAAAACGACGCGAACG3′; unmethylated primers, F:5′GTTTTGTAGTTTTTGGAGTTAGTGTTGTGT3′ and R:5′CTCAACCTACAATCAAAAACAACACAAACA3′. All polymerase chain reactions (PCRs) were performed using TaKaRa EpiTaq HS (for bisulfite-treated DNA, TaKaRa Bio, Mountain View, CA, USA): 1XEpiTaq PCR Buffer (Mg^2+^ free), 2.5 mM MgCl_2_, 0.3 mM dNTPs, 20 pmol of each primer (Sigma-Aldrich), 50 ng of DNA, and 1.5 U of TaKaRa EpiTaq HS DNA Polymerase in a 50 µL final reaction volume. PCR cycling conditions were as follows: initial denaturation at 95°C for 30 sec, followed by 35 cycles consisting of 95°C for 30 sec, the respective annealing temperatures for 30 sec, and 72°C for 30 sec, followed by a final extension at 72°C for 7 min. Methylated *SFRP1* promoter region was amplified at the annealing temperature of 65.5°C, while unmethylated region was amplified at 63.1°C. PCR products were separated and visualized on 2% agarose gels stained with GelStar (Lonza Rockland, Inc. Rockland, ME, USA). Positive control for methylated reaction was Methylated Human Control (Promega) and that for unmethylated reaction were human white blood cell DNA and ovary. Negative control was nuclease-free water.

### Immunohistochemistry

The samples were fixed in formalin, embedded in paraffin, sliced into 4-μm thick sections, and mounted into capillary gap microscope slides (DakoCytomation, Glostrup, Denmark). Sections were immunostained using streptavidin horseradish peroxidase/DAB (Dako REAL EnVision Detection System Peroxidase/DAB+, Rabbit/Mouse, Dako) as described previously (37). The primary antibodies used were as follows: SFRP1 (rabbit polyclonal anti-human; Clone: sc-13939, Santa Cruz Biotechnology, Santa Cruz, CA, USA, dilution 1:200), beta-catenin (mouse monoclonal anti-human beta-catenin antibody; Dako Corporation, Carpinteria, CA, USA, dilution 1:200), TCF1 (mouse monoclonal anti-human TCF1; Clone A-79, dilution 1:50), and LEF1 (Clone, REMB1; Santa Cruz Biotechnology, Dallas, TX, USA, dilution 1:50) applied for 30 min at room temperature. Negative controls underwent the same staining procedure but the samples were not incubated with primary antibodies. Positive controls were the frontal cortex of the normal adult brain, human placenta, and normal human ovary tissues. Immunohistochemical analysis was evaluated by three independent observers in a blinded fashion, who had no knowledge on gene status, tumor stage, and demographic information. Cells in the hot spot, an area containing the most characteristics of malignant tissue and most active proliferative rate, of each sample were analyzed. The staining intensity was scored by comparing immunoreactivity to normal tissue expression level and by using 10% and 50% cut-offs as follows: 0/+: no reactivity or few focally weakly positive cells (<10% tumor cells); ++: heterogeneous moderate reactivity staining (10 to 50% tumor cells); +++: homogeneous intense reactivity (>50% tumor cells).

### Quantitative stereological analysis of SFRP1 protein in brain tumor tissue

Quantitative stereological analysis of volume density (Vv) was performed by Nikon Alphaphot binocular light microscope (Nikon, Vienna, Austria) using Weibel’s multipurpose test system with 42 points (M 42) at 400 × magnification (34). The area tested was 0.0837 mm^2^, and the total length of the lines was 1.008 mm. For each investigated group the orientation/pilot stereological measurement was carried out to define the number of fields to be tested. The volume density of SFRP1-positive cells was determined according to the point- counting method. Volume density was calculated using the formula Vv = Pf/Pt, where Pf is the number of hits test points on SFRP1-positive cells and Pt is the number of all test points in the tested area.

### Statistical analysis

Sample size was determined based on tumor incidence, financial considerations, and other studies of similar size in the investigated field. *Post-hoc* power analysis was performed regarding our primary aim. The normality of data was tested using the Shapiro-Wilk test. Pearson χ^2^ and Spearman’s correlation were used to test the relationships between *SFRP1* hypermethylation and protein expression level, SFRP1 localizations, beta-catenin, TCF1 and LEF1, grades, and other clinical and demographic features. Significance level was set at *P* < 0.05. Statistical analysis was performed using Statistica, version 13.0 (Dell, Tulsa, OK, USA, licensed to the School of Medicine, University of Zagreb).

## Results

### *SFRP1 *promoter methylation

**Figure 1:**
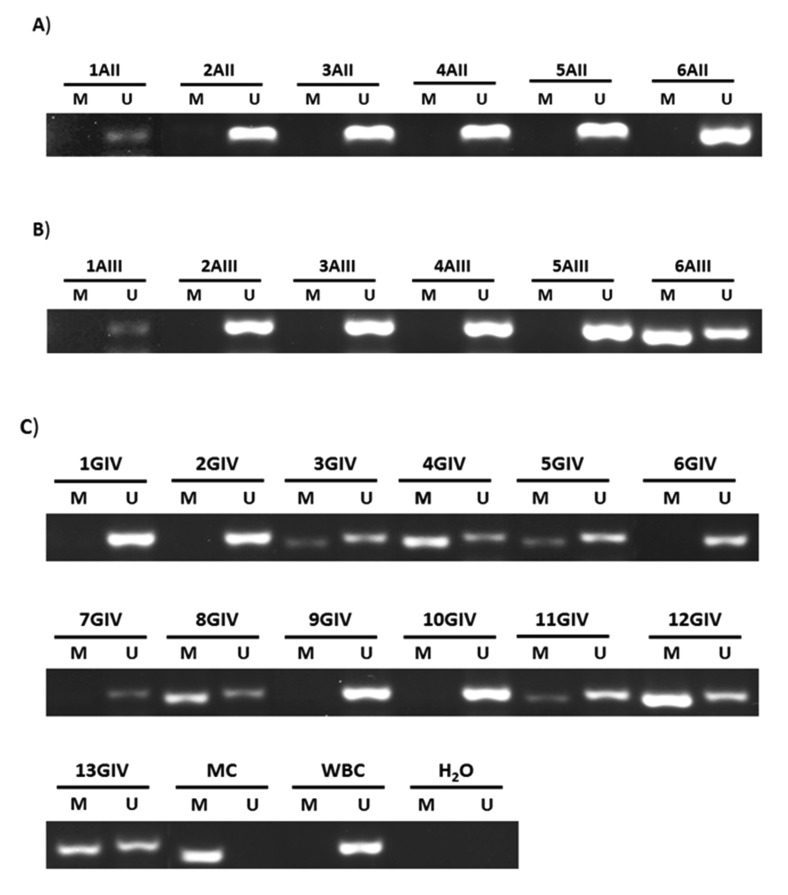
Methylation-specific polymerase-chain-reaction analysis for *Secreted Frizzled Related Protein 1* (*SFRP1*) gene promoter in astrocytic brain tumors (**A**) grade II (1AII-6AII); (**B**) grade III (1AIII-6AIII); and (**C**) grade IV (1GIV-13GIV). The presence of a visible polymerase chain reaction product (band) in gel lanes marked U indicates the presence of unmethylated promotors; the product presence in lanes marked M indicates the presence of methylated promotors. Methylated human control (MC) was used as positive control for methylated reaction, human white blood cell DNA (WBC) was used as positive control for unmethylated reaction, and water was used as negative control.

Epigenetic analysis found 32% (8/25) of methylated brain tumors. Malignant astrocytomas of different grades had very different methylation patterns of *SFRP1* gene promoter. All diffuse astrocytomas showed an unmethylated pattern (Figure 1A). Also, the majority of anaplastic astrocytomas (5/6 or 83.3%) showed an unmethylated pattern (Figure 1B). In the remaining one anaplastic astrocytoma sample, both gel bands were visible, the band demonstrating the unmethylated promoter and the band demonstrating the methylated promoter, but the band demonstrating methylated promoter was stronger. There were 7 of 13 glioblastoma samples with methylated *SFRP1* promoter (53.9%) (Figure 1C). All the methylated samples also had bands demonstrating unmethylated promoter, but 3 of them had considerably stronger methylated bands. The remaining 46.2% (6/13) of glioblastomas had an unmethylated promoter without any trace of methylation (Table 1). When we compared *SFRP1* methylation status among tumor grades, a significant difference in methylation distribution was revealed (Pearson χ^2^ = 6.323; *P* = 0.042). Methylation of *SFRP1* promoter appeared more frequently in glioblastoma than in other astrocytoma grades, which was confirmed by Spearman’s correlation analysis (Spearman’s rho 0.502; *P* = 0.011).

**Table 1:** Promoter methylation status, expression levels, and localizations of Secreted Frizzled Related Protein 1 (SFRP1), beta-catenin, lymphoid-enhancer factor 1 (LEF1), and T-cell factor 1 (TCF1) proteins in astrocytoma samples, and demographic and clinical data of astrocytoma patients

WorldHealth Organization grade	No. of patients	*SFRP1* promoter methylation status*	Levels of expression^†^				Sex	Age	Intracranial localization^‡^
			SFRP1	beta-catenin	LEF1	TCF1			
AII (diffuse astrocytoma)	1	U	3	1			M	56	temporal R
	2	U	3	3			M	48	frontal R
	3	U	3	3			F	38	occipital R
	4	U	1	2	2	1	M	49	frontal R
	5	U	1	1	2	1	M	44	insular L
	6	U	1	2	1	2	M	32	temporal L
AIII (anaplastic astrocytoma)	1	U	2	2			F	55	frontal L
	2	U	3	3			M	58	frontal R
	3	U	3	3			M	46	frontal L
	4	U	1	2	2	1	F	34	frontoparietal parasagittal R
	5	U	1	2	3	3	M	24	frontal L
	6	**M**/U	2	2	2	1	M	51	frontal parasagittal L
G IV (glioblastoma)	1	U	1	1	3	2	M	68	parietal R
	2	U	1	3	2	1	M	56	frontal R
	3	M/**U**		1	2	1	F	54	temporooccipital R
	4	**M**/U	2	2			F	62	parietal R
	5	M/**U**		1			F	58	temporoparietal L
	6	U	1	1	3	2	F	77	temporal L
	7	U	1	1	3	3	M	60	temporal L
	8	**M**/U	1	3	3	1	F	56	parietal L
	9^§^	U	2	2	3	3	M	31	temporal L
	10	U	3	3	1	1	F	71	frontal L
	11	M/**U**	3	1			F	74	frontal R
	12	**M**/U	1	2	3	3	F	56	temporal R
	13^II^	M/U	2	1	3	3	M	38	temporooccipital R
	14^II^		2	1	3	3	M	54	frontal R

*M – methylated; U – unmethylated; bold – pronouncedly present, regular – present.

†1 – weak or lack of expression; 2 – moderate expression; 3 – strong expression.

‡R – right; L– left.

§Patients with *isocitrate dehydrogenase 1 *mutation.

IIPatients with *ATRX Chromatin Remodeler* mutations.

### SFRP1 protein expression and its association to DNA methylation

Aberrant methylation alters gene expression. Therefore, we were interested in the expression levels of SFRP1 protein and its subcellular localization. The tissue expression levels were determined by the semiquantitative method in the 3-stage signal strength and by quantitative stereological analysis. The results obtained by two methods of signal quantification were strongly positively correlated (Spearman’s rho 0.518; *P* = 0.010).

**Figure 2:**
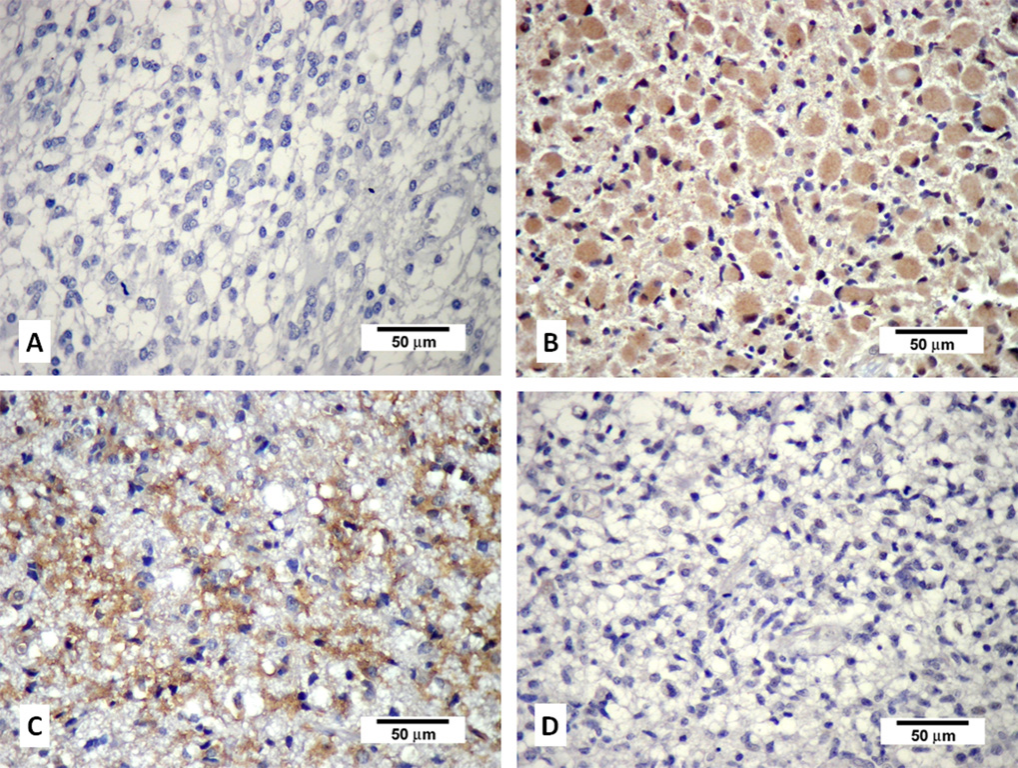
Characteristic immunohistochemical staining of Secreted Frizzled Related Protein 1 (SFRP1) expression in astrocytoma. (**A**) Negative control; (**B**) astrocytic brain tumor grade II showing strong cytoplasmic staining; (**C**) astrocytic brain tumor grade IV (glioblastoma) showing strong cytoplasmic staining (both **B** and **C** with unmethylated promoters of *SFRP1* gene); (**D**) astrocytic brain tumor grade IV (glioblastoma) with methylated promoter of *SFRP1* gene showing lack of expression.

In order to assess signal expression levels of SFRP1, the immunostains were compared to the expression levels of normal control tissues, which showed strong or moderate SFRP1 expression levels. Of total astrocytoma samples, 45.8% (11/24) had weak or lack of SFRP1 protein expression, 25% (6/24) had moderate, and 29.2% (7/24) had strong expression. Subcellular distribution of SFRP1 protein was predominantly cytoplasmic, with only 20.8% (5/24) of samples showing simultaneous nuclear immunostain. Expression levels, when distributed to different grades, were not associated to any particular grade (Pearson χ^2^ = 2.218; *P* = 0.330). Samples with methylated *SFRP1* showed significantly lower SFRP1 protein expression than samples with unmethylated *SFRP1* (Pearson χ^2^ = 8.874; *P* = 0.031) (Figure 2B-D). Ratios of investigated analyses are shown in Table 2.

**Table 2:** *Secreted Frizzled Related Protein 1 *(*SFRP1*) promoter methylation status, expression level of SFRP1, beta-catenin, lymphoid-enhancer factor 1 (LEF1), and T-cell factor 1 (TCF1) proteins in astrocytoma samples

	World Health Organization grade*								
	A II (diffuse astrocytoma)			A III (anaplastic astrocytoma)			G IV (glioblastoma)		
	U	M/U	M/U	U	M/U	M/U	U	M/U	M/U
**Methylation status of SFRP1 promoter**	6/6			5/6		1/6	6/13	3/13	3/13 1/13‡
**Expression status†**	**1**	**2**	**3**	**1**	**2**	**3**	**1**	**2**	**3**
**SFRP1**	3/6		3/6	2/6	2/6	2/6	6/12	4/12	2/12
**Beta-catenin**	2/6	2/6	2/6		4/6	2/6	8/14	3/14	3/14
**LEF1**	1/3	2/3			2/3	1/3	1/11	2/11	8/14
**TCF1**	2/3	1/3		2/2		1/3	4/11	2/11	5/11

*M – methylated; U – unmethylated; bold – pronouncedly present; regular – present.

†1 – weak or lack of expression; 2 – moderate expression; 3 – strong expression.

‡Sample with same band intensities.

### Activation of Wnt signaling in glioblastoma

We investigated if *SFRP1* methylation and protein expression affected the activation/inactivation of Wnt signaling. Therefore, we investigated expression levels and subcellular localization of beta-catenin, whose up-regulation and transfer to the nucleus indicates Wnt activation. In 61.5% (16/26) of astrocytomas beta-catenin expression was up-regulated (denoted as moderate and strong) as compared to weak expression observed in normal brain tissue, while in 57.7% (15/26) beta-catenin was found in the nucleus. In the glioblastoma group, this localization was even more pronounced (9/14; 64.3%). The majority of samples with unmethylated or predominantly unmethylated *SFRP1* promoter showed low beta-catenin expression levels. Statistical analysis confirmed this observation, showing that glioblastomas with unmethylated *SFRP1* promoter had significantly less beta-catenin protein (Pearson χ^2^ = 4.550; *P* = 0.033). In glioblastomas with strongly methylated *SFRP1,* beta-catenin was up-regulated and transferred to the nucleus.

Up-regulation of transcriptional activators reveals the activation of Wnt signaling. Therefore, we tested the correlation between LEF1 and TCF1 expression levels and *SFRP1* methylation pattern. Although 52.9% (9/17) and 41.2% (7/17) of total astrocytomas showed strong and moderate expression levels of LEF1 and TCF1, respectively, there was no correlation to methylation profile or to SFRP1 expression: LEF1 and methylation (Spearman’s rho = 0.079; *P* = 0.781), TCF1 and methylation (Spearman’s rho = -0.084; *P* = 0.766), LEF1 and SFRP1 expression (Spearman’s rho = 0.046; *P* = 0.867), TCF1 and SFRP1 expression (Spearman’s rho = 0.211; *P* = 0.433). However, strong LEF1 expression levels were significantly correlated with higher astrocytoma grades (Spearman’s rho 0.642, *P* = 0.006).

### Association to epidemiological characteristics

We also investigated the association of epidemiological characteristics of our astrocytoma samples to *SFRP1* methylation status and expression levels of the proteins. Men had significantly more unmethylated tumors than women (Pearson χ^2^ = 4.588; *P* = 0.043). No significant association was observed between SFRP1 expression levels and sex. To investigate the differences between the patients' age and methylation and expression patterns we divided our sample into two age groups: younger than 55 and older than 55. Age was not associated to methylation (Pearson χ^2^ = 1.186; *P* = 0.276), but 5 out of 8 methylated cases were in the >55 group. Age was also not associated to SFRP1 expression. Both groups showed similar levels of SFRP1 expression.

The presence of *IDH1 *and *ATRX* mutations was also tested. Although *IDH1* and *ATRX* mutation positivity was not significantly associated to the methylation profile of *SFRP1*, all mutated cases had strong LEF1 and TCF1 expression levels, and for TCF1 this association was marginally significant (Pearson χ^2^ = 4.950; *P* = 0.061). Beta-catenin expression was not associated to the mutation presence.

## Discussion

Our study showed that 32% of astrocytoma samples had hypermethylated promoter, and that the number of such samples increased from grade II to higher astrocytomas grades. Our hypothesis was affirmed and the results suggest that SFRP1 is involved in the progression of these tumors. As shown by methylation-specific PCR, all of diffuse astrocytomas had unmethylated *SFRP1* promoter, while 16.7% of anaplastic and 53.9% of glioblastomas had methylated promoter. Glioblastomas were significantly more methylated than lower astrocytoma grades. A given locus can have a different DNA methylation status because of extensive cell-to-cell and tissue heterogeneity. In cancer DNA, both bands can be visible and have different intensities, since tumors consist of heterogeneous cells, some containing a methylated and some an unmethylated gene. Furthermore, the transition from unmethylated to methylated status of a gene in a specific group of tumor cells can be discrete.

Glioblastoma subtypes differ, among other things, in DNA methylation patterns of specific genes (6,38). There are genes which are present in the minority of one tumor type, but when they are present, their methylation status makes a considerable difference. Alterations of DNA methylation of cancer-causing genes can change the expression of such genes and result in transformation or progression of cancer cells (39). Furthermore, methylation status can also have predictive significance. For instance, patients with methylated *O6-methylguanine-DNA-methyltransferase* gene promoter benefit from alkylating agent chemotherapy.

Although in our total sample there was 45.8% of astrocytomas with weak or lack of SFRP1 protein expression, 25% with moderate, and 29.2% with strong expression, the observed expression values were not significantly assigned to any specific grade. However, we found that methylated cases expressed significantly less SFRP1 protein than unmethylated. This result is in accordance with the role that has been assigned to SFRP1 in many cancers (40), including glioma: a missing SFRP1 cannot interact with Wnt proteins, which enables Wnts to bind to frizzled receptors and activate the pathway.

Several articles indicated that Wnt signaling was involved in glioblastoma invasion (15-17,41-44) and tumor cell migration (13,45). Furthermore, many authors have demonstrated that SFRP1 suppresses tumor growth and inhibits Wnt signaling (46,47). Majchrzak-Celińska et al (32) showed that gliomas were characterized by aberrant promoter hypermethylation of many Wnt pathway antagonists, including *SFRP1*. Their findings are similar but not identical to ours. They detected methylated tumors in all three grades, while we did not detect methylated grade II tumors and detected only one methylated grade III tumor. Also, they analyzed different types of glial tumors, while we focused only on astrocytic type. Nevertheless, they (32) also concluded that *SFRP1* methylation was more frequent in patients with tumor grades III and IV, which is consistent to our finding. Foltz et al (30) indicated that histone modification was responsible for the epigenetic modulation of Wnt antagonist, showing that DKK1, SFRP1, and WIF1 had decreased expression in human glioblastoma.

Götze et al (1) found frequent promoter hypermethylation of several Wnt pathway inhibitor genes. Their results on *SFRP1* across astrocytoma grades are similar to ours: grade III samples harbored 14.3% and grade IV 53.3% of methylated promoters. They also reported lower SFRP1 expression in the methylated than in unmethylated samples, but on the mRNA level.

SFRPs are soluble proteins that modulate Wnt signaling (48,49). Although SFRPs family members were generally attributed with antagonistic mode of action (50,51), their action need not always be inhibitory (52,53). There is accumulating evidence that specific SFRP members could also promote tumorigenesis, ie, act as agonists of Wnt signaling depending on the cellular and functional context (54). Our previous investigations on SFRP3 in astrocytomas (53) revealed complex expression patterns distinct from the present findings on SFRP1 expression. In the present study, we clearly demonstrated that this member of the SFRP family acted as a classical tumor suppressor in astrocytoma biology. A recent comprehensive study on the context-specific roles of SFRPs (47) analyzed promoter methylation, gene expression, and survival data from 8000 tumors of 29 cancer types, finding that only SFRP1 consistently functioned as a tumor suppressor.

The Wnt pathway is activated through its main effector molecule, beta-catenin (23,41). We showed that glioblastoma samples with unmethylated *SFRP1* promoter had significantly less beta-catenin protein. In the samples with strongly methylated *SFRP1* promoter beta-catenin was up-regulated and transferred to the nucleus. Several studies including our previous research (55-58) showed that in glial tumors beta-catenin was up-regulated and expressed in the nucleus. The present study confirmed these findings but also presented a link between epigenetic silencing of *SFRP1* and beta-catenin expression. The following scenario could explain our findings: since methylated samples failed to synthesize SFRP1 protein, there is no antagonistic activity upon Wnt signaling and the oncogenic beta-catenin levels are rising. Similar results were obtained by Chang et al (59), who found a lower SFRP1 expression rate in glioblastoma than that in the normal brain and inversely correlated protein levels of SFRP1 and beta-catenin. They also showed that positive SFRP1 expression was associated with longer overall survival rates. Kierulf-Vieira et al (60) showed that treatment with recombinant SFRP1 protein in primary glioma stem cell cultures down-regulated nuclear beta-catenin and decreased *in vitro* proliferation. Schiefer et al (31) studied the *SFRP* gene family in glioblastoma cell lines by using a different approach – inducing DNA demethylation by inhibiting DNA methyltransferases.

We found no significant association of transcription activators of Wnt signaling, LEF1 and TCF1, to methylation profile or to SFRP1 expression levels. This could be explained by the small sample size or by beta-catenin’s ability to switch co-transcriptional partners (61). Astrocytic tumors, especially glioblastoma, harbor great heterogeneity. The invasive potential of glioblastoma tumor cells in the brain parenchyma is tremendous. There was, however, a correlation between strong LEF1 expression and higher astrocytoma grades.

Several novel articles indicate that SFRP1 is a good candidate for a therapeutic target (43,62-64). Recently the anti-proliferative activity of purified SFRP1 was demonstrated in two cancer cell lines (65), which is important for the development of pharmaceutical interventions.

Our study is limited by small number of samples, which diminishes the statistical power and might have prevented us from detecting some possibly existing true associations, while the associations that reached significance might have been inflated. *Post-hoc* power analysis regarding our primary aim showed that comparison of *SFRP1* methylation frequency between glioblastoma and other tumor types had 46.7% power. Therefore, our results should be interpreted with caution before they are independently reproduced. Further studies with a larger sample size are needed to elucidate the precise mechanism by which SFRP1methylation affects glioma biology and the potential associations to prognosis.

Since biological and clinical behaviors of astrocytoma could only partially be explained by morphology, it is important to search for and define novel molecular biomarkers. Our results indicate that SFRP1 is involved in the progression of astrocytic glial tumors, offering a potential methylation biomarker.
